# Effect of transcranial direct current stimulation on sports performance for two profiles of athletes (power and endurance) (COMPETE): a protocol for a randomised, crossover, double blind, controlled exploratory trial

**DOI:** 10.1186/s13063-020-04412-0

**Published:** 2020-06-03

**Authors:** Yohan Grandperrin, Sidney Grosprêtre, Magali Nicolier, Philippe Gimenez, Chrystelle Vidal, Emmanuel Haffen, Djamila Bennabi

**Affiliations:** 1 Service de Psychiatrie de l’Adulte, Centre Hospitalier Universitaire de Besançon, 25030 Besançon Cedex, France; 2 Laboratoire de Neurosciences Intégratives et Cliniques EA481, Université de Bourgogne Franche-Comté, 19 rue Ambroise Paré, 25000 Besançon, France; 3 Laboratoire Culture, Sport, Santé, Société EA 4660, Université de Bourgogne Franche –Comté, UPFR Sports, 25000 Besançon, France; 4 Centre d’Investigation Clinique, INSERM CIC 1431, Centre Hospitalier Universitaire de Besançon, 25030 Besançon Cedex, France; 5 Centre Expert Dépression Résistante FondaMental, Centre Hospitalier Universitaire de Besançon, 25030 Besançon Cedex, France

**Keywords:** Transcranial direct current stimulation, Endurance performance, Explosive performance, Dorsolateral prefrontal cortex, Primary motor cortex, Parkour, Cycling

## Abstract

**Background:**

Transcranial direct current stimulation (tDCS) is promising for improving motor and cognitive performance. Nevertheless, its mechanisms of action are unclear and need to be better characterised according to the stimulated brain area and the type of exercise performed.

**Methods/design:**

This is a double-blind crossover study, organised into two parts: the first is to assess the effects of tDCS on explosive performance (jump task) and the second is to assess the effects on endurance performance (cycling time trial task). Participants, who are recreationally active or athletes (parkour practitioners, cyclists), will receive two active tDCS sessions (over the left dorsolateral prefrontal cortex and right motor cortex) and one sham tDCS session (part A), or two sequences (one active and one sham) of two daily tDCS sessions over 5 days (part B). Motor and cognitive performance will be compared before and after tDCS sessions (part A), and before and after the first session, after the last session and at day 12 and day 30 of each tDCS sequence (part B).

**Discussion:**

This study investigates the acute and repeated effects of tDCS on the motor and cognitive performance of healthy subjects. It will try to evaluate if tDCS could be considered as a neuroenhancement technology according to the physical task investigated (endurance versus explosive).

**Trial registration:**

ClinicalTrials.gov, NCT03937115. Registered on 3 May 2019; retrospectively registered.

## Background

Over the past decade, neurostimulation techniques have been used for improving cognitive and psychomotor functions in healthy subjects. Among them, transcranial direct current stimulation (tDCS) is a safe, low-cost, portable, non-invasive neuromodulation technique that delivers low-intensity, direct current to cortical areas. tDCS induces changes in cortical excitability that can last from a few minutes to several hours after the stimulation [1, 2].

In addition, tDCS could also improve exercise performance and reduce neuromuscular fatigue. A recent meta-analysis by Machado et al. assessed the tDCS effects on performance improvement during different exercises (muscle strength exercise or whole body dynamic cyclic exercise) [3]. Eleven studies (*N* = 236 participants) were included in this quantitative analysis. The authors found weak evidence of a beneficial effect of anodal tDCS applied over the primary motor cortex (M1) before the cycling time to exhaustion, while cathodal stimulation had no detrimental effect on cycling performance. It should be noted that these results are strongly influenced by a single study. Moreover, anodal tDCS would not have any effect on the isometric strength of the upper or lower limbs and few studies have evaluated the effects of tDCS on isokinetic or dynamic muscle strength [3]. To sum up, many studies have demonstrated a positive enhancement of performance using tDCS [4–6] while others failed to find any improvement [7–9]; therefore, no particular consensus could be made based on the literature. Several factors may explain such a disparity in these results, from the type of physical or cognitive task used to the montage of the electrodes.

First, tDCS was used to enhance different types of exercise (e.g., isometric, dynamic or isokinetic strength, cycling) where the intensity could vary according to the protocol (e.g., cycling time to exhaustion, trial cycling time). According to Angius et al. [10], it is necessary to separate studies with single-joint exercise and whole-body exercise due to differences in the cardiorespiratory, metabolic, and neuromuscular responses [11]. It also seems appropriate to separate endurance and explosive tasks. Basically, explosive efforts require the production of maximum power output over a minimum amount of time, while endurance tasks require effort management to maintain a targeted submaximal level of performance over a long period. tDCS could, thus, improve motor performance by different ways depending on the type of exercise practised: increase in the maximal power output, decrease in the perceived exertion, or modulation of pacing strategies.

Second, the discrepancy of the stimulation’s parameters—electrode placement, current intensity, density, tDCS timing (online versus offline)—from one study to another could explain the conflicting results obtained in previous literature. Accordingly, tDCS configuration seems to be a major factor to explore. Angius et al. have compared cephalic—the anode over the left M1 and the cathode over the right dorsolateral prefrontal cortex (dlPFC)—and extracephalic—the anode over the left M1 and the cathode over the shoulder—tDCS montages and have found an improvement of the isometric endurance performance of the lower limb with the extracephalic montage. Changes in the current direction and negative effects of the return electrode would explain the differences between the two montages [5].

The mechanisms of tDCS action, therefore, remain unclear. Since they could widely depend on the stimulated brain region, two areas attract our attention: the M1 and the dlPFC. Regarding endurance tasks, neuromuscular fatigue is multifaceted and influenced by both central and peripheral factors. Peripheral fatigue results in changes at or distal to the neuromuscular junction whereas central fatigue represents the inability of the central nervous system (CNS) to generate or maintain central activation of the muscle. Central fatigue is accompanied by changes in the activity of the spinal motoneurons (spinal fatigue) and a reduction in the motor cortical neuronal drive (supraspinal fatigue) [12, 13]. If tDCS over the M1 modulates the corticospinal output [14], it could increase the excitability of the motor areas and delay the detrimental effects of fatigue over the neural drive of active muscles. In regards to those considerations, the prefrontal cortex (PFC) could also be a target to improve performance. In fact, the PFC increases neuronal activation to reinforce muscle force during an exercise [15]. It may have a motivational function and a role in pacing strategies [16, 17]. tDCS over the PFC could improve motivation and inhibit or reduce negative external factors (e.g., muscle pain), leading to a gain in performance. More particularly, due to the key role of the left dlPFC in cognitive control, decision-making and approach motivation, and the benefit of tDCS effects on cognitive function, this latter appears as the main area of interest [18–21]. Since the excitability of both of these areas could theoretically be modulated to increase maximal power output, the question of the response to tDCS in terms of explosive performance remains open. In fact, despite rare clues in the literature showing a positive effect on vertical jump performance [22], no study really assessed tDCS effects over such type of exercise and its associated mechanisms. Indeed, when it comes to tDCS effects on physical tasks, the literature is more developed regarding endurance performance.

To clarify the effect of tDCS on these different types of neuromuscular performance, we propose to evaluate the acute and repeated effects of tDCS during two tasks: an explosive task (jumps) and an endurance task (20-min cycling trial). The main objective is to compare the effects of tDCS applied over the left dlPFC with sham tDCS on the performance of the neuromuscular system. In the present protocol, the choice has been made to recruit participants with different backgrounds regarding sports practice, from sedentary people to high-level athletes in the targeted performance (parkour athletes for explosive performance or cyclists for endurance). Indeed, the tDCS effects on physical performance could also be a function of the initial level [23].

This study is a monocentre, sham-controlled, randomised, crossover, double-blinded superiority trial comparing active tDCS versus sham tDCS. It is divided into two parts: part A with the jumpers and part B with the cyclists. We hypothesise that the active tDCS sessions will improve motor performance and reduce neuromuscular fatigue. We will seek a better understanding of the factors that could cause tDCS effects to vary: stimulated brain area, exercise type (explosive vs enduring), tDCS configuration (cephalic vs extracephalic montage), and the number of tDCS sessions (single vs repeated stimulations).

## Methods/design

### Study setting and overview

This research will be carried out through a collaboration between the Psychiatric Department of the University Hospital of Besançon and the scientists from the Faculty of Sports of the University of Besançon (C3S Laboratory). All experiments will take place on the EPSI research platform (Entraînement Performance Santé Innovation, Besançon, France). This protocol will be divided into two parts to explore the effects of tDCS by type of exercise:

- Part A, which assesses the effects on explosive performance (jumps)
- Part B, which assesses the effects on endurance performance (cycling time trial)

Fifty subjects (20 in part A, 30 in part B) will be recruited from the university or from federated organisations of sports. For part A, athletes practicing parkour, an activity that consists of jumping obstacles in various environments, have been chosen since they usually present a very explosive neuromuscular profile [24]. For part B, amateur and professional cyclists will be recruited. After information about the study, written informed consent will be obtained.

**Fig. 1:**
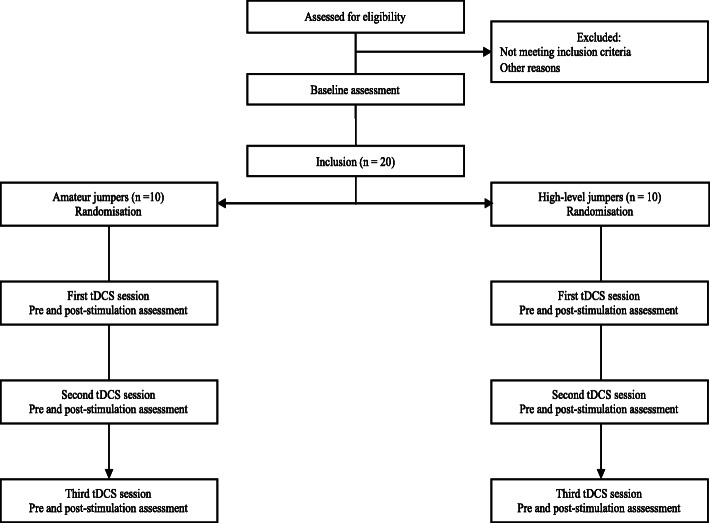
Study flow diagram (part A)

In part A, subjects will be divided into two groups (amateur vs high-level practice) and receive three sessions of tDCS (active over the left dlPFC vs active over the right M1 vs sham over the left dlPFC). The session order will be randomised (Fig. 1).

**Fig. 2:**
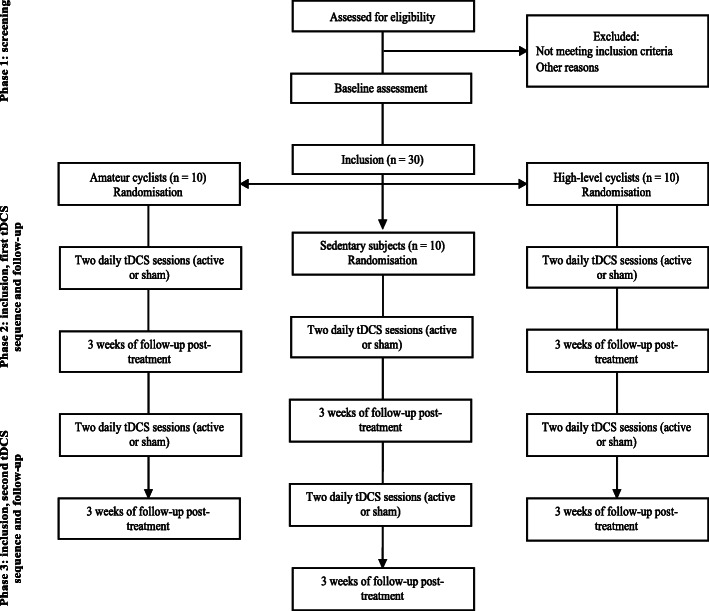
Study flow diagram (part B)

In part B, subjects will be divided into three groups (sedentary, amateur, and high-level practice). They will receive two sequences of tDCS divided into two daily active tDCS sessions (over the dlPFC) over 5 days and after a wash-out of one month, two daily sham tDCS sessions over 5 days, or vice and versa (Fig. 2).

Common measurements for parts A and B will include: clinical assessment of impulsivity based on self-report scales and behavioural tasks, task-based measures of motivation, and assessment of neuromuscular function—electromyographic (EMG) recordings and evoked potentials from nerve percutaneous stimulation. These measures will be taken before and after each brain stimulation session (part A) or before and after each brain stimulation sequence, and at day 12 and day 30 (part B).

For each group, baseline measures will include a clinical assessment of depression severity, based on the Quick Inventory Depression Scale-Clinician version (QIDS-C16) and on the self-reported version (QIDS-SR16). These data will be compared to those obtained after the last tDCS session and at days 12 and 30 (part B).

In order to assess the tDCS effects on two types of physical performance, the physical tasks will differ between parts A and B. In part A, the performances assessed will be jumping tasks—squat jump (SJ), countermovement jump (CMJ), and standing long jump (SLJ)— while in part B, an endurance task (20 min cycling time trial) will be performed. The endurance task will be performed maximally pre- and post-tDCS sequence, then at day 12 and day 30, while it will be performed sub-maximally (60% of peak power) during each online tDCS training session (i.e., twice a day for 5 days). In part B, other measures will be performed at the start, immediately after the last session of tDCS and then at day 12 and day 30 (see “Study procedure” section for details). After unblinding, active and sham stimulation outcomes will also be compared.

### Inclusion criteria

Eligible subjects will be invited to take part in this trial according to the following criteria: (1) subjects over 18 years old; (2) right-handed; (3) no addictive comorbidities (except tea, coffee, tobacco) and no severe progressive neurologic and/or somatic and/or psychiatric disease; (4) part A: amateur jump practice (less than 4000 h of practice, which represents for example 15.5 h of training per week during the last 5 years) or high-level jump practice (more than 4000 h of practice); OR (5) part B: amateur cycling practice (less than 4000 h of practice) or high-level cycling practice (more than 4000 h of practice), or sedentary (less than 2 hours of recreational sports practice per week).

The training volume of 4000 h to identify highly trained participants has been established according to the literature on young high-level athletes. A “high level of specialisation” in a given activity has been defined for example as experience of ~ 7 years at 11 h of training per week averaged over a year [25], which represent a total of 4000 h. The training volume was fixed in total number of hours rather than the number of years and training frequency, since an athlete can also reach more than 16 h of training per week at the peak of their career. Therefore, 4000 h can be achieved in 5 years at 15.5 h per week, this latter scenario being given as an example.

### Exclusion criteria

Subjects will be excluded if they are identified as having any of the following: (1) younger than 18 years of age; (2) left-handed; (3) presence of psychiatric or addictive diseases; (4) presence of severe somatic or progressive neurologic pathologies; (5) low cooperation stated by the investigator; (6) pregnancy; (7) concurrent participation in another trial; (8) no coverage by the national health insurance; and (9) measure of protection or under guardianship of justice.

### Interventions

#### Transcranial direct current stimulation

Direct current will be delivered by a neurostimulator system (StarStim®, Neuroelectrics©, Barcelona, Spain) that allows a sham double-blind mode. It will be transmitted by two saline-soaked synthetic sponge electrodes (Sponstim®, 25cm^2^) placed in a neoprene head cap.

In part A, subjects will benefit from three 20-min sessions of tDCS (two active and one sham), separated by a minimum of 48 h. Electrodes will be placed according to the EEG 10–20 International System (Table 1) and sequence order will be determined by computer randomisation.

**Table 1:** Electrode placement and stimulation parameters in part A

Part A	Sequence order determined by randomisation		
Anode	F3 over left dlPFC	FC_2_ over right motor cortex	Sham: F3 over left dlPFC
Cathode	AF8 over right supraorbital region	Controlateral shoulder	AF8 over right supraorbital region
Intensity of stimulation	2 milliAmpers	2 milliAmpers	0 milliAmper

In part B, subjects will benefit from two sequences of 20-min tDCS sessions per day, for 5 days consecutively (either a sequence of ten active or ten sham sessions according to the computer randomisation). After a wash-out of one month, they will receive the second sequence (crossover). The anodal electrode will be placed over F3 (left dlPFC) and cathodal electrode over AF8 (right supraorbital region). The intensity of stimulation will be 2 mA (in active sequence) or 0 mA (sham sequence).

For sham stimulation, in each part of the protocol, the current will gradually ramp up over 30 s until 2 mA at the beginning of the stimulation and will then gradually ramp down over an equal amount of time at the end, thus leading to the same initial and final sensations of active tDCS.

#### Neuromuscular assessment

The general performance of the neuromuscular system will be assessed on plantar flexors. Participants will be seated in a comfortable chair in a relaxed position. They will be instructed to keep their hands free and particular care will be taken so that the trunk stays against the chair back.

EMG activity of plantar-flexor muscles will be recorded continuously during motor tasks (maximal voluntary contractions, jumps, and cycling time trial). EMG activity will be recorded from four muscles of the right leg: soleus (SOL); medial gastrocnemius (MG); tibialis anterior (TA); vastus lateralis (VL). Before electrode placement, the skin will first be shaved and dry-cleaned with alcohol to keep low impedance (< 5 kΩ). The EMG signal will be recorded with Trigno sensors (Delsys, Natick, MA, USA). The sensors will be firmly strapped to the leg with skin rubber and placed according to the SENIAM recommendations [26]. EMG signals will be amplified with a bandwidth frequency ranging from 0.3 Hz to 2 kHz (gain 1000) and digitised on-line (sampling frequency 2 kHz) with Labchart software (LabChart 8, ADInstruments, Sydney, Australia). The root mean square (RMS) value of SOL, GM, GL, TA, and VL muscle EMG signals will be determined with an integration time of 500 ms over the plateau during plantar flexion maximal force, prior to the stimulus artefact for trials with electrical stimulations. SOL, MG, GM, and RMS will be normalised by the corresponding maximal muscle compound action potential recorded during maximal force production [M_SUP_].

Neuromuscular function will be assessed by means of recording motor potentials evoked on triceps surae muscles by peripheral nerve electrical stimulations. The nerve-evoked potentials will be elicited to account for the relative contributions of the several nervous levels (at the neuromuscular junction, at spinal and at supraspinal levels) to the possible changes induced by tDCS. The evolution of these evoked potentials following acute or chronic interventions is commonly assessed to account for neuromuscular changes, particularly at the spinal level [27].

The posterior tibial nerve will be stimulated through single rectangular pulses (1-ms width) delivered by Digitimer stimulators (model DS7A, Hertfordshire, UK). Stimulations will be elicited with a self-adhesive cathode (8-mm diameter, Ag-AgCL) placed in the popliteal fossa, and an anode (5 × 10 cm, Medicompex SA, Ecublens, Switzerland) placed over the patella. The monitoring of TA EMG activity during the setting of the stimulation electrode will ensure that the common peroneal nerve will not be activated. Three different responses will be recorded and taken for analysis: the H-reflex, the maximal muscle compound action potential (M-wave), and the V-wave. The H-reflex is a classic tool to investigate spinal excitability by reflecting the efficiency of Ia-to-alpha motoneuronal transmission. The V-wave characterises the magnitude of the neural drive from M1 addressed to the spinal motoneuronal pool. The aim of recording the maximal M-wave is twofold: it serves as a marker of the excitability of the neuromuscular junction; and it is used to normalise each of the other responses.

At rest, the stimulation intensity will be first progressively increased from SOL and GM responses’ threshold with 2 mA increments to obtain maximal H-reflex (H_MAX_) and then with 5 mA increments until the M-wave of triceps surae muscles no longer increases. This last stimulation intensity will then be increased by 20% to ensure supramaximal stimulation and used to record maximal M-wave (M_MAX_). Three intensities will be identified: the one that gives 50% of the H_MAX_ in the ascending phase of the H-reflex recruitment curve (H_50_), H_MAX_, and M_MAX_. At muscle level, M_MAX_ characterises the direct activation of the muscle at the neuromuscular junction while at the spinal level, H_50_ and H_MAX_ reflect spinal Ia-to-alpha motoneuronal transmission. Four stimulations will be performed at each intensity to obtain PRE measurements.

With those stimulation parameters, stimulations at maximal H-reflex and M-waves will also be superimposed to maximal voluntary contractions (MVC), H_SUP_ and M_SUP_, respectively. It can be noticed that M_SUP_ is followed by a V-wave, which is used as an index of the supra-spinal descending neural drive [28]. To perform MVCs and record plantar flexor force, the ankle will be firmly strapped to a pedal equipped with a constraint gauge (PCE Instruments, France). Participants will be asked to focus on plantar flexion, avoiding any other unnecessary movement. The recording of one antagonist (TA) and one knee extensor (VL) will allow us to minimise the contribution of other muscle groups to the developed force. Participants will be asked to perform four MVCs of 4 s (two for H_SUP_ and two for M_SUP_), during which stimulations will be manually triggered during the force plateau. MVCs will separated by a minimum of a 1-minute rest.

The mechanical signals will be digitised online (sampling frequency 2 kHz) and simultaneously recorded with electromyography of the targeted muscles. Signals will be stored for analysis in Labchart software (LabChart 8, ADInstruments, Sydney, Australia).

Peak-to-peak amplitudes of electromyographic responses at rest (H_50_, H_MAX_, M_MAX_) and during MVC (H_SUP_, M_SUP_, V) will be measured for quantitative analysis. It can be noticed that maximal H-reflex, reflecting spinal excitability, is generally associated with a small M- wave (MatH at rest and MatHsup during MVC), which will also be measured. For each muscle, all responses will be normalised to maximal M-wave evoked in the same condition. Thus, H_50_/M_MAX_, H_MAX_/M_MAX_, M_atH_/M_MAX_, H_SUP_/M_SUP_, M_atHsup_/M_SUP_, and V/M_SUP_ will be considered as dependent variables.

In part A, each variable of the neuromuscular assessment will be performed before and after each tDCS session. In part B, it will be performed before and after the first sequence of tDCS, then at day 12 and day 30 for each sequence of the crossover.

#### Explosive or endurance task

In part A, three types of jump will be performed by the subjects, before and after each tDCS session. After a standardised warm-up (jumping on the spot, running, knee raises, etc), subjects will benefit from several trials per jump. The order of the jumps will be randomised.

Horizontal jump performance will be characterised by the standing long jump (SLJ), performed on a graduated anti-slip mat. Participants will be allowed to perform SLJ until performance no longer increases, with 20 to 30 s rest between each trial. The maximal metered performance is measured in centimetres from the front edge of the force platform to the rear part of the most indented heel.

Vertical jumps will be performed on a force plate (Kistler, Winterthour, Switzerland), with continuous recording of vertical ground reaction force at a sampling frequency of 1000 Hz. Two types of maximal vertical jump will be performed: squat jump (SJ) and counter movement jump (CMJ). Participants will be asked to jump and land with both feet simultaneously on the force plate, with no initial steps or shuffling. Angles of the knee and ankle will be visually controlled during all landings. The SJ will be assessed from a starting position with knees flexed at 90° and weight well distributed over both feet. Participants will be asked to keep their trunk straight, and no counter movement with the legs is allowed. For the CMJ performance, participants will begin in a standing upright position. They will be asked to bend to 90° knee flexion and immediately jump without pausing in the squat position. For both SJ and CMJ, participants will keep their hands on their hips. Suspension time of vertical jumps will be measured and the vertical jump performance measured in centimetres.

In part B, the endurance task will be performed maximally pre- and post-tDCS sequence, then at day 12 and day 30 of each sequence while it will be performed sub-maximally (at 60% of maximal power) during each tDCS training session (i.e., twice a day for 5 days). Maximal power of each subject will be determined during the first cycling time trial (day 1).

The endurance task will consist of a pedalling task on a cycloergometer at constant frequency (70 rpm) at a fixed duration of 20 min, corresponding to a usual time trial in cycling competitions. Participants will be asked to provide the maximal output during the whole duration of the test. They will be able to modulate by themselves the resistance of the pedals by turning a wheel located on the handlebars.

During this effort, no feedback will be displayed. Subjects will not have access to the effort-related parameters (speed, distance covered, etc.) except for the time remaining. During the performance, different measures will be continuously recorded, such as myoelectrical activity, power output, heart rate, and pedalling rate. Every 2 minutes, participants will be asked to give their rate of perceived exertion (Borg CR10 scale) and their rate of pain perception (Cook scale).

An habituation session will be conducted at the time of inclusion.

#### Cognitive tasks

The cognitive tasks will be common to both parts of the study.

The tDCS effects on delay discounting will be assessed using the Monetary Choice Questionnaire (MCQ) [29]. The MCQ is a task composed of 27 items of hypothetical monetary choices between smaller immediate rewards and larger delayed ones. The rewards can be small, medium, or large and vary from 11 to 85€. The delays vary between 7 and 186 days. The calculation of delay discounting is based on the hyperbolic function V = A/ (1 + *k*D). V represents the subjective value of the delayed reward, A is the amount of the delayed reward, D is the delay, and *k* is the coefficient that estimates the subjective discounting rate for the given delayed reward. *K*-values will be generated by the 27-item MCQ Automated Scores [30] for overall discounting rates of each subject and will be compared before and after the tDCS session (jumpers) or before and after the first and the last tDCS session and at day 12 and day 30 of each sequence (cyclists).

The tDCS effects on different aspects of impulsivity will be assessed using four tasks: the French version of the Barratt Impulsiveness Scale (BIS-10), the experimental Go/No-Go and Stroop tasks, and the Balloon Analog Risk Task (BART).

The BIS-10 is a 34-item self-report questionnaire that measures overall and specific impulsivity (cognitive, motor, and non-planning impulsivity) [31]. Each item is rated 0, 1, 3, or 4 points and the overall scores of impulsivity thus vary from 0 to 136.

The Go/No-Go task is issued from the Frontal Assessment Battery [32]. The subjects must inhibit a response that was previously given to the same stimulus (e.g., not tapping when the examiner taps twice), in order to assess their difficulties in controlling impulsivity. The scores range from 0 to 3 depending on the number of errors.

The Stroop task is issued from the GREFEX battery [33]. This task is divided into three parts: a naming task (where the subject quotes colours as quickly as possible), a reading task (where the subject reads the name of the colours as quickly as possible), and an interference task (where the subject names the colour they observe and not the one that is written as quickly as possible). The interference is obtained by subtracting the denomination time from the interference time.

The BART task is a computer-based measure of the risk-taking [34]. During this task, the subjects must press a button to inflate a series of 30 balloons displayed on the computer. Each pump corresponds to 5 cents, which are accumulated in a temporary bank (the amount of which is unknown to the subject). At any time, the subjects can collect the money obtained in a definitive bank (the amount of which is displayed on the computer). However, if the balloon explodes before collection, the money accumulated in the temporary bank is lost and a new balloon appears on the screen. Each balloon has a different probability of explosion and the subject’s objective is to make as much money as possible. Risk-taking behaviour will be measured by the adjusted average number of pumps (only trials in which the balloons did not explode are included in the calculations).

The tDCS effects on motivation will be measured using the Effort Expenditure for Rewards Task (EEfRT) [35]. It is a computerised effort-based decision-making task. For each trial, the subject must choose between an easy and a difficult task.

During the easy task, the subject must press the “L” key on the keyboard with the index finger of the right hand and can earn 1€ according to the probability of retribution. During the hard task, the subject must press the “S” key with the pinkie of the left hand and can earn between 1.24 and 4.30€ (“reward magnitude”) according to the probability of retribution. The probability of reward retribution varies at each trial; an indication of this probability (12%, 50%, or 88%) is given before the choice. The subject thus makes a choice between easy and difficult tasks according to the probability of reward and the reward magnitude. Motivation is modelled by the percentage of hard task choices.

All of these tasks will be performed before and after each tDCS session (jumpers), or before and after the first and the last tDCS session, and at day 12 and day 30 of each sequence (cyclists).

A comparison of inter-condition scores and scores before and after tDCS session/sequence will be made.

Finally, depression severity will be assessed by the clinician (QIDS-C16) and self-reported by the subject (QIDS-SR16) [36].

The QIDS-SR16 has 16 items (score range from 0 to 27) assessing the severity of depressive symptoms as perceived by the subject, with the following cut-off points: 0–5 none; 6–10 mild; 11–15 moderate; 15–20 severe; and 21–27 very severe depression.

The QIDS-C16 has 16 items (score range from 0 to 27) assessing the severity of depressive symptoms as perceived by the psychiatrist, with the same cut-off points.

These depression scales will be applied at participant’s inclusion and after the last session or sequence of tDCS.

#### Pointing task

In part A, evaluation of the acute effect of tDCS on fine motor performance will also be performed, by means of a visual pointing task. In a sitting position, participants will have to point with a pencil (dominant hand) between two targets as accurately and as fast as possible. The targets are black squares designed on paper displayed on the table in front of the participant. The distance between participants’ trunk and the table and targets will be kept constant between each measurement. The targets will be displayed in a frontal axis, with the nearest target aligned with the shoulder and the furthest target shifted by 45° on the left. Three different levels of target difficulty will be set according to target widths and distances between them. Three widths (W = 0.5, 1.5, and 4 cm) and three centre-to-centre target distances (D = 15, 20, and 35 cm) will be used to manipulate the index of difficulty (ID), calculated by the formula: ID = log2(2D/W). One trial will consist of five cyclical pointing movements as accurately and as fast as possible between two targets of the same size, namely ten arm movements, always starting and finishing on the nearest target. The total time to perform these ten movements is taken into account for each trial. Two trials will be performed per ID (total of six trials). Subjects will have to perform the pointing task under real conditions but also to imagine themselves performing these six trials. They will be particularly instructed to feel pointing between the targets (kinesthetic imagery) as they would actually do [37]. They will be asked not to track the targets visually. The comparison of imagined and real trials therefore provides different clues about the different stages of the movement, from movement planning to programming, and execution of the considered task [38].

### Outcomes

Our efficacy criteria will be the evaluation of neuromuscular performance during an explosive task (main outcome, part A) or during an endurance task (secondary outcomes, part B).

In part A, we will compare the height of the vertical jumps (in centimetres) or the length of horizontal jumps (in centimetres) performed before and after active or sham tDCS session. In part B, endurance performance will be assessed by comparing the average power output (in watts) during a time-trial realised before, after the first, on the last tDCS session, and at day 12 and day 30 of each sequence.

Secondary efficacy criteria include:

- tDCS effects on the neuromuscular system by analysis of the EMG signals during MVC, evoked potentials (muscle and spinal excitability, voluntary activation), and comparison of the results obtained before and after tDCS session (jumpers) or before and after the first and the last tDCS session, and at day 12 and day 30 of each sequence (cyclists)
- tDCS effects according to the motor expertise by comparison of performance (in centimetres for vertical jump heights and horizontal jump lengths) between amateur subjects and those with high-level jump practice, or sedentary and amateur subjects and those with high-level cycling practice
- tDCS effects on motor gestures and speed–accuracy trade-offs during a pointing task before and after the tDCS session (jumpers), by analysing times (in seconds) to complete the different pointing tasks
- Changes in the rating of perceived exertion and muscle pain by comparison of the scores from the Borg CR10 scale (from 1 to 10) and the Cook’s scale (from 1 to 10) obtained during the endurance task (every two minutes) before and after the first and the last tDCS session and at day 12 and day 30 of each sequence (cyclists)
- Changes in motivation, by comparison of scores from the EEfRT (from 0 to 100) obtained before and after the tDCS session (jumpers) or before and after the first and the last tDCS session and at day 12 and day 30 of each sequence (cyclists).
- tDCS effects on impulsivity by comparison of the scores from BIS-10 (from 0 to 136), the experimental Go/No-Go (from 0 to 3), Stroop (interference score) tasks, and the BART (adjusted average number of pumps) obtained before and after the tDCS session (jumpers) or before and after the first and the last tDCS session, and at day 12 and day 30 of each sequence (cyclists)
- Changes in delay discounting, by comparison of the scores from the MCQ (from 0.00016 to 0.24,942) obtained before and after the tDCS session (jumpers) or before and after the first and the last tDCS session and at day 12 and day 30 of each sequence (cyclists)
- Changes in depression severity, by comparison of scores from the QIDS-SR16 and QIDS-C16 (from 0 to 27) obtained at the inclusion and after the last tDCS session (jumpers) or the last tDCS sequence (cyclists)

### Study procedure

#### Recruitment and randomisation

Firstly, subjects will be recruited by the sport research team. Information about the study, the neurostimulation technique, and the objectives of the research will be given to each subject by a trained psychiatry investigator. Enrolment date and timetable of visits will be scheduled directly with the volunteers.

After the informed consent is signed, a clinical exam will be conducted in order to verify the inclusion and exclusion criteria.

Subjects meeting the inclusion criteria will be randomised 1:1 into two (part A) or three (part B) groups using a minimisation technique with stratification according to their athletic level (hours of practice). Recruitment will be achieved when the number of subjects by groups is obtained.

After randomisation, a sequence of predefined codes will be generated by a computer. These predefined codes correspond to either the active or sham stimulation and will be used by the psychiatry staff to start the stimulator, allowing a double-blind study design. Each subject will present a control subject with the same randomisation sequence.

#### Blinding

Patients, researchers, and medical staff will be blind to the allocation to either active or sham stimulation. A computer-generated predefined code will be used to start the computer program connected by Wi-Fi to the stimulator. Correspondence between the codes and the type of stimulation will only be available after unblinding at the end of the study.

#### Study procedure: Part A

**Fig. 3:**
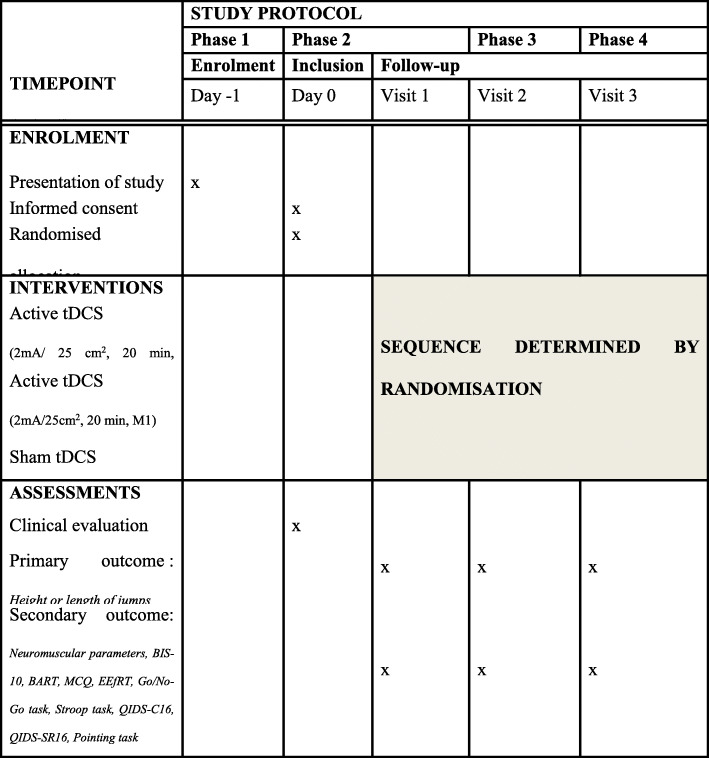
Randomised crossover design for COMPETE (part A) (Standard Protocol Items: Recommendations for Interventional Trials (SPIRIT) Figure). *BART* Balloon Analog Risk Task*, BIS-10* Barratt Impulsiveness Scale-10, *dlPFC* dorsolateral prefrontal cortex, *EEfRT* Effort Expenditure for Rewards Task, *M1* primary motor cortex, *MCQ* Monetary Choice Questionnaire, *QIDS-C16* 16-Item Quick Inventory of Depressive Symptomatology, Clinician Rating, *QIDS-SR16* 16-Item Quick Inventory of Depressive Symptomatology, Self-Report

In part A, the study will have four phases. The first phase will correspond to the recruitment while other phases will correspond to both inclusion of volunteers and visits for the experimental sessions. The three visits will be organised in the same way and separated by a wash-out of at least 48 h. This period will be necessary to allow a wash-out of the effects of a tDCS session, which persist for several hours [1]. Cognitive and motor tasks and neuromuscular assessments will be realised before and immediately after the stimulation. The detailed procedure is displayed in Fig. 3.

#### Study procedure: Part B

**Fig. 4:**
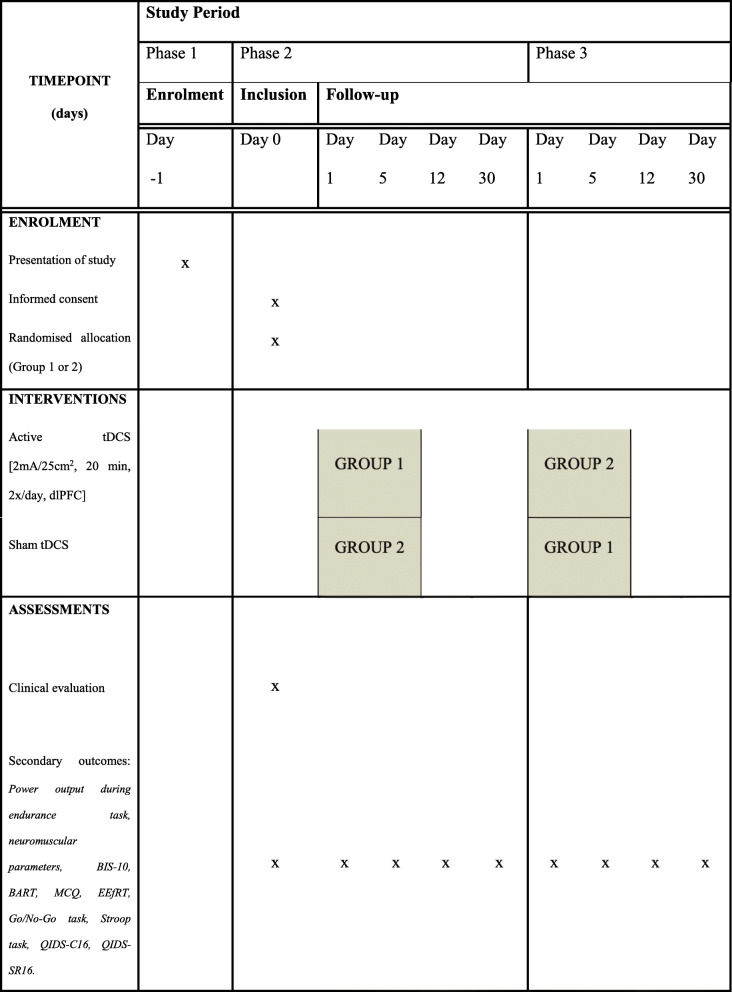
Randomised cross-over design for COMPETE (part B) (Standard Protocol Items: Recommendations for Interventional Trials (SPIRIT) Figure). *BART* Balloon Analog Risk Task, *BIS-10* Barratt Impulsiveness Scale-10, *dlPFC* dorsolateral prefrontal cortex, *EEfRT* Effort Expenditure for Rewards Task, *M1* primary motor cortex, *MCQ* Monetary Choice Questionnaire*, QIDS-C16* 16-Item Quick Inventory of Depressive Symptomatology, Clinician Rating*, QIDS-SR16* 16-Item Quick Inventory of Depressive Symptomatology, Self-Report

In part B, the study will be comprised of three phases (the detailed procedure is displayed in Fig. 4). The first phase will correspond to recruitment, conducted in the same way as in part A. The second phase will relate to both the inclusion of the volunteers and the period of the first tDCS sequence. Behavioural scores and neuromuscular parameters will be collected at baseline and immediately at the first tDCS session. It will be delivered on a Monday and two daily sessions will be performed during the following days (during the endurance task) up to Friday. Clinical, neuromuscular, and behavioural assessments will be realised once the last tDCS session has been delivered (day 5), and then at day 12 and day 30.

After the last assessment, crossover will be realised: subjects who underwent sham stimulation sessions will then be submitted to active sessions and vice versa (third phase) with the same design. One month of wash out will be necessary to eliminate the residual effects of the tDCS sequence.

### Sample size

Our sample size calculation is based on the primary efficacy outcome that relates to changes in motor performance before and after a tDCS session during an explosive task (jumps). In a previous study, Lattari et al. [22] showed an 11.2% improvement in the height of the CMJ following a tDCS session, corresponding to a difference of 3.9 cm (Table 1 of Lattari’s et al.). We expect a 15% improvement in our study corresponding to a difference of 5.1 cm. No difference is expected in the sham tDCS group. Considering a significance level of 5%, a power of 90%, and a standard deviation for paired differences of 6.5 (calculated from standard deviations, page 21, Table 1 with an hypothesis of a covariance of measures of 50%), 20 jumpers are included to meet the objectives of the study. Sample size calculation was performed on PASS 13 Power Analysis and Sample Size Software (2014) [39, 40] .

### Withdrawal of consent

Participants will be informed that taking part is completely voluntary and that they are free to withdraw from the study at any time without prejudice and without having to give a reason. They may also be removed at any time from the study if adverse events or any exclusion criteria are detected. If a disease is discovered during the study, subjects will be offered a medical follow-up adapted to it. Withdrawals of consent will be replaced.

### Data management and statistical analyses

All collected information will be registered in physical files (case report files (CRFs)), previously anonymised with the participant’s randomisation code in order to respect confidentiality at all times. Computer test data (BART, EEfRT) and physical test results will be collected in electronic format.

All researchers and trained staff called upon to collaborate in the tests are bound to secrecy.

Analyses will be performed using the SAS® 9.4 Software for Windows (SAS Institute©, Cary, NC, USA). Categorical variables will be described in terms of effective, absolute, and relative frequencies for each modality. Continuous variables will be described in terms of minimum and maximum, quartiles, means, and standard variations. Two-way repeated measures ANOVAs will be performed with the within-group factors of condition (a-tDCS/ c-tDCS/sham-tDCS) and moment (pre- and post-tDCS) for endurance performance or explosive performance. Bonferroni corrections will be employed to correct for type I errors due to multiple testing. The sphericity assumption will be tested using the Mauchly’s test and the Greenhouse-Geisser correction will be used whenever data sphericity is violated. The level of significance will be set at *p* ≤ 0.05. Subgroup analysis (level of practice) will be descriptive because of the small sample size of the study. Means and proportions will be calculated with their 95% confidence interval. All valid data will be used at different times of the study. There is no strategy for replacing missing data.

### Monitoring

COMPETE is a project classified in category 2 of the French Jardé Law and is approved by the Committee for the Protection of Persons. This classification does not require a data monitoring committee. Data monitoring will be conducted by the University Hospital of Besançon, in accordance with the French legislation and the European Medicine Agency’s Guideline on Data Monitoring Committees. Monitors will have documented competence to follow up the research and no competing interests. Monitoring visits to the centre will take place annually to verify adequate progress of the research and respect for ethical regulations. Investigators will store all administrative documents, patient identification logs, signed patient consent forms, copies of the data documentation forms, and common study documentation. Original data of study subjects will also be stored. A list allowing patient identification will be kept for 15 years (Directive 2001/83/EG). The investigator should retain the study documents for at least 15 years after the completion or discontinuation of the clinical study.

Any adverse events occurring after the consent signing will be reported to the requesting authority.

### Ethics and dissemination

The study is prospectively registered on ClinicalTrials.org as “Effect of tDCS on Sport Performance for Two Categories of Athletes: Explosive Profile and Enduring Profile”, identifier NCT03937115 (available at https://clinicaltrials.gov/ct2/show/NCT03937115). This protocol is approved by the French Committee for the Protection of Persons Est IV, under the number 18/47. It adheres to the Standard Protocol Items: Recommendations for Interventional Trials (SPIRIT) guidelines (Additional file 1: SPIRIT Checklist—COMPETE).

Prior to enrolment, the principal investigator will provide full information about the study to the volunteers. If they agree to participate, they will sign written informed consent. Subjects will be informed that taking part in the study is completely voluntary, and that they are free to withdraw from the study at any time without prejudice and without having to give a reason.

Data management and monitoring respect the French Jardé Law (No. 2012–300, from 5 March 2012) and the French Public Health Code’s guidance on good clinical practice to conduct trials of human participants.

Dissemination will be provided by the research team through presentations at conferences and scientific publications.

## Discussion

COMPETE is an ambitious protocol that will seek to understand the acute and long-term effects of tDCS on physical performance. It will compare the tDCS effects on two types of exercise (explosive vs endurance) using a different administration mode (single vs repeated sessions).

The challenge of this study will be to specify the action mechanisms of tDCS on performance according to the exercise type, the stimulated brain area, the tDCS configuration type, and the level of athletes included.

To the best of our knowledge, this study will be the first to assess the effect of tDCS on different sport performance by gathering psychometric and neuromuscular measurements. It will determine whether and how the improvement or disruption of the cognitive dimensions studied (mood, motivation, and impulsivity) could affect explosive and/or endurance performance.

Finally, some authors have already argued that tDCS may be considered as a new form of doping. Many athletes use tDCS during training for several hours [41], although the effects of prolonged administration are unclear. Our study could determine if chronic use involves risks for them and raise the question of legislation around the free use of tDCS.

### Trial status

The study is recruiting subjects from November 2018 until April 2021 and aims to enrol 50 subjects. This protocol is version 2.0, 29 May 2018.

## Supplementary information

**Additional file 1.** SPIRIT 2013 Checklist: Recommended items to address in a clinical trial protocol and related documents.

## Data Availability

Data sharing is not applicable to this article as datasets were not generated during the current study.

## Abbreviations

BART: Balloon analog risk task
BIS-10: Barratt Impulsiveness Scale-10
CMJ: Counter movement jump
CNS: Central nervous system
CRF: Case report form
dlPFC: Dorsolateral prefrontal cortex
EEfRT: Effort expenditure for rewards task
EEG: Electroencephalography
EMG: Electromyography
H_Max_: Maximal H-reflex
H_SUP_: Maximal H-reflex superimposed to MVC
ID: Index of difficulty
M1: Primary motor cortex
MCQ: Monetary Choice Questionnaire
MG: Medial gastrocnemius
M_MAX_: Maximal M-wave
Msup: Maximal M-wave superimposed to MVC
MVC: Maximal voluntary contraction
PFC: Prefrontal cortex
QIDS-C16: Quick Inventory of Depressive Symptomatology, Clinician Rating-16 items
QIDS-SR16: Quick Inventory of Depressive Symptomatology, Self-Report-16 items
RMS: Root mean square
SJ: Squat jump
SLJ: Standing long jump
SOL: Soleus
TA: Tibialis anterior
tDCS: Transcranial direct current stimulation
VL: Vastus lateralis
